# Essential Role of STAT3 Signaling in Hair Follicle Homeostasis

**DOI:** 10.3389/fimmu.2021.663177

**Published:** 2021-11-11

**Authors:** Kosuke Miyauchi, Sewon Ki, Masao Ukai, Yoshie Suzuki, Kentaro Inoue, Wataru Suda, Takeshi Matsui, Yoshihiro Ito, Kenya Honda, Haruhiko Koseki, Osamu Ohara, Reiko J. Tanaka, Mariko Okada-Hatakeyama, Masato Kubo

**Affiliations:** ^1^ Laboratory for Cytokine Regulation, Center for Integrative Medical Science (IMS), RIKEN Yokohama Institute, Yokohama, Japan; ^2^ Laboratory for Integrated Cellular Systems, Center for Integrative Medical Science (IMS), RIKEN Yokohama Institute, Yokohama, Japan; ^3^ Graduate School of Medical Life Sciences, Yokohama City University, Yokohama, Japan; ^4^ Department of Computer Science and Systems Engineering, Faculty of Engineering, University of Miyazaki, Miyazaki-shi, Japan; ^5^ Laboratory for Microbiome science, Center for Integrative Medical Science (IMS), RIKEN Yokohama Institute, Yokohama, Japan; ^6^ Graduate School of Frontier Sciences, The University of Tokyo, Chiba, Japan; ^7^ Laboratory for Evolutionary Cell Biology of the Skin, School of Bioscience and Biotechnology, Tokyo University of Technology, Hachioji, Japan; ^8^ Laboratory for Gut Homeostasis, Center for Integrative Medical Science (IMS), RIKEN Yokohama Institute, Yokohama, Japan; ^9^ Department of Microbiology and Immunology, Keio University School of Medicine, Tokyo, Japan; ^10^ Disease Biology Group, RIKEN Medical Sciences Innovation Hub Program, Kanagawa, Japan; ^11^ Laboratory for Developmental Genetics, Center for Integrative Medical Science (IMS), RIKEN Yokohama Institute, Yokohama, Japan; ^12^ Laboratory for Integrative Genomics, Center for Integrative Medical Science (IMS), RIKEN Yokohama Institute, Yokohama, Japan; ^13^ Department of Applied Genomics, Kazusa DNA Research Institute, Kisarazu, Japan; ^14^ Department of Bioengineering, Imperial College London, London, United Kingdom; ^15^ Institute for Protein Research, Osaka University, Suita-shi, Japan; ^16^ Division of Molecular Pathology, Research Institute for Biomedical Science, Tokyo University of Science, Noda-shi, Japan

**Keywords:** STAT3 (signal transducer and activator of transcription 3), skin, atopic dermatitis, bacteria, hair follicle (HF)

## Abstract

Dominant-negative mutations associated with signal transducer and activator of transcription 3 (STAT3) signaling, which controls epithelial proliferation in various tissues, lead to atopic dermatitis in hyper IgE syndrome. This dermatitis is thought to be attributed to defects in STAT3 signaling in type 17 helper T cell　specification. However, the role of STAT3 signaling in skin epithelial cells remains unclear. We found that STAT3 signaling in keratinocytes is required to maintain skin homeostasis by negatively controlling the expression of hair follicle-specific keratin genes. These expression patterns correlated with the onset of dermatitis, which was observed in specific pathogen-free conditions but not in germ-free conditions, suggesting the involvement of Toll-like receptor-mediated inflammatory responses. Thus, our study suggests that STAT3-dependent gene expression in keratinocytes plays a critical role in maintaining the homeostasis of skin, which is constantly exposed to microorganisms.

## Introduction

Signal transducer and activator of transcription 3 (STAT3) signaling plays a critical role in maintaining stem cell pluripotency and ensuring stem cell survival (1, 2), and it is associated with many aspects of cell differentiation (3–6). STAT3 is a major signal transduction/transcription factor involved in diverse processes, including wound healing, angiogenesis, immune responses, nervous system development, and cancer (7–9). Hyper IgE syndrome (HIES) results from a dominant-negative *STAT3* gene and is a primary immune deficiency characterized by atopic dermatitis (AD)-like dermatitis (10–12). Defects in type 17 helper T cell (T_H_17) specification have been considered the major cause of HIES, which has been attributed to decreased expression of retinoid-related orphan receptor (ROR)-γt, a protein that is induced by the interleukin (IL)-6 and IL-23 receptors (13, 14). Partial improvements in dermatitis in HIES patients treated with hematopoietic stem cells have suggested that the role of STAT3 signaling in cells other than T cells is involved in the onset of dermatitis (15). However, the roles of STAT3 signaling in skin homeostasis remain unknown.

It has been shown that STAT3 signaling is required for skin wound healing, keratinocyte migration, and hair follicle (HF) growth (16, 17). The topical application of JAK inhibitors has been observed to trigger hair telogen-to-anagen transition, suggesting the importance of JAK/STAT signaling in the development of HF stem cells (HFSCs) (18). STAT3 is reported to play a role in HF formation and the hair cycle during the second anagen phase (19, 20), and HF-derived cytokines regulate the trafficking of dendritic cells to the skin and the maintenance of resident T cells (21, 22). Constitutive expression of the active form or the deletion of STAT3 in the basal layer of HFs suggests an essential role in keratinocyte stem/progenitor cell homeostasis (23, 24). The skin epidermis is a stratified epithelium that forms a barrier to protect against mechanical stress and infections. The epidermis undergoes constant turnover, and keratinocyte stem/progenitor cells are responsible for homeostasis of the epidermal compartments. In the present study, we attempted to understand the role of STAT3 signaling in the homeostasis of epidermal compartments.

Epithelial keratins are classified as acidic type I and basic or neutral type II and are coexpressed as specific pairings with one of each type. Keratin polymerization occurs based on α-helical rod domains in type I and type II keratins to form heterodimers that are arranged in parallel. Further polymerization of these heterodimers builds intermediate filaments that comprise the cytoskeleton of epithelial cells (25). Mutations in the type I and type II keratin proteins that form heterodimers cause an imbalance in the epidermal compartments, leading to several skin diseases (26–28). However, little is known about how STAT3 contributes to strict regulation of the specific pairing of keratin proteins.

In the present study, we directly investigated the contribution of STAT3 to skin homeostasis and dermatitis by generating mice with conditional deficiencies in this protein in the skin. Our data clearly indicate that STAT3 signaling is required to maintain the homeostatic expression pattern of type I and II HF-specific keratin genes under specific pathogen-free (SPF) conditions. Furthermore, dysregulation of the homeostatic expression of HF-keratin genes in infant skin were determined to be associated with Toll-like receptor (TLR)-mediated inflammatory responses in this STAT3 mouse model of AD. We further discuss how STAT3 signaling during early life influences the regulation of skin homeostasis.

## Results

### STAT3 Signaling Controls Gene Expressions of HF-Specific Keratins

**Figure 1:**
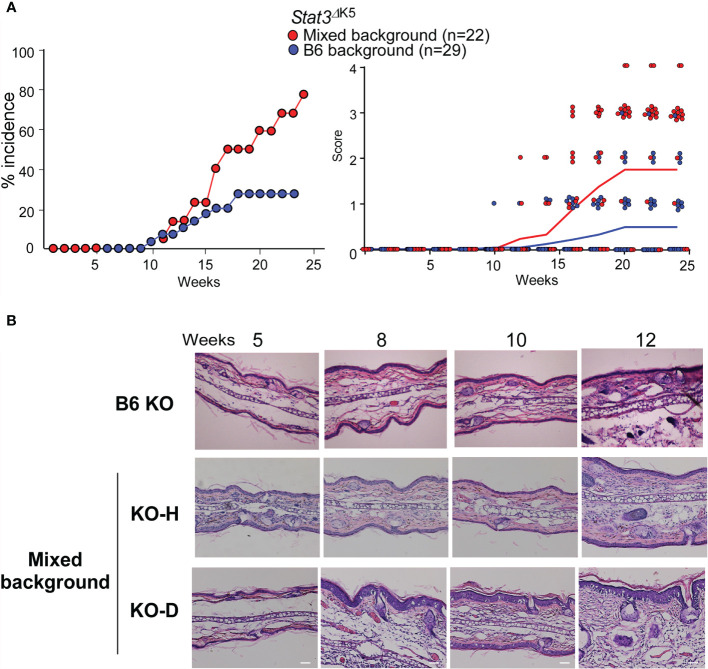
Atopic dermatitis (AD)-like dermatitis frequently develops in mixed-background *Stat3* ^ΔK5^ mice. **(A)** Percentage of mixed- or B6-background *Stat3* ^ΔK5^ mice with skin lesions at indicated weeks of age (left). The clinical scores were analyzed for mixed (n=22) or B6 (n=29) background *Stat3* ^ΔK5^ mice at the indicated weeks of age (right). Line colors indicate means of clinical scores in each group. Clinical scores were determined as follows: 0, no lesion; 1, lesion on periocular area; 2, lesion on part of the face; 3, lesion on the whole face; 4, lesion on ear. **(B)** Skin hematoxylin and eosin staining images of non-onset B6 and mixed background healthy (KO-H) or onset (KO-D) *Stat3* ^ΔK5^ mice at indicated weeks of age. Scale bar = 100 μm.

To investigate how STAT3 signaling contributes to the development of AD-like signs, we examined the effects of STAT3 loss-of-function in the skin of *Stat3* ^ΔK5^ mice in a mixed 129×C57BL/6 background (mixed) and a pure C57BL/6 background (B6). *Stat3* ^ΔK5^ mice of both genetic backgrounds developed AD signs. The mixed mice exhibited >80% of AD signs, whereas disease onset in B6 mice was reduced by 20% (**Figure 1A**, left), and the disease score was milder in the B6 mice than in the mixed mice (**Figure 1A**, right). The *Stat3* ^ΔK5^ mice were divided into two groups as follows: healthy individuals with no dermatitis on the face until 20 weeks (KO-H), and diseased mice with onset by 20 weeks (KO-D). Histological analysis based on hematoxylin and eosin (HE) staining demonstrated that the mixed KO-D model mice displayed atypical dermatitis at 10 weeks of age (**Figure 1B**). These results indicate that STAT3-mediated signaling has an essential but partial role in disease onset.

**Figure 2:**
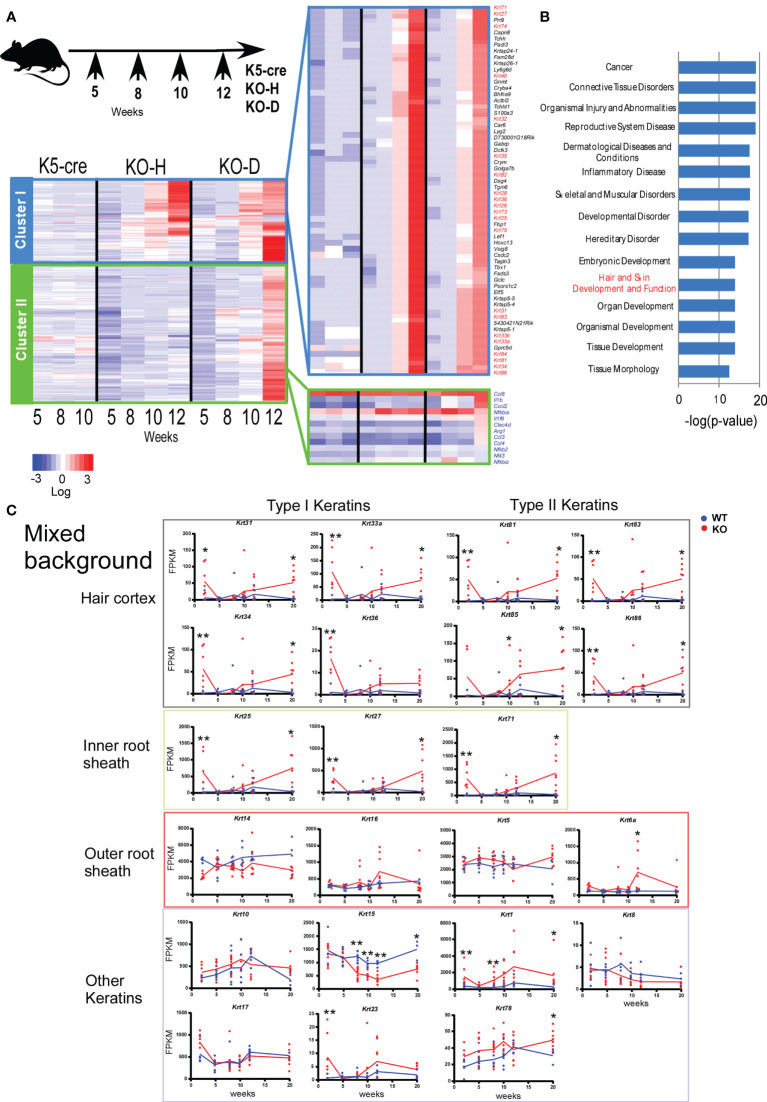
Clustering of gene expression profiles of ear skin from K5-cre and *Stat3* ^ΔK5^ mice. **(A)** *Stat3* ^ΔK5^ mice were divided into two groups based on disease onset as follows: healthy with no dermatitis until 20 weeks (KO-H) and disease onset mice (KO-D) (top). Total RNA was isolated from the ears of individual mice, specifically K5-cre (n=2), KO-H (n=5), and KO-D (n=6), at 5, 8, 10, and 12 weeks of age and analyzed by RNA-seq. The 2,064 differentially expressed genes (DEGs) in control and *Stat3*-deficient cells (p<0.05, FPKM>2.0) were extracted by comparing the expression values among the K5-cre, KO-H, and KO-D groups at each indicated week, and clustering analysis was performed using Ward’s method based on the chronological change. The gene groups belonging to the two clusters were determined by gene expression changes over time. Finally, expression values of the genes in each cluster were aligned in the graph for visualization as heat maps. Keratin genes are indicated with red. Genes associated with Toll-like receptor (TLR) –NF-κB pathways are indicated with blue. **(B)** The top 15 ranking pathways in the Ingenuity Pathway Analysis (IPA) of DEGs in cluster I are shown with p-values. **(C)** The expression profiles of hair follicle (HF)-specific or other keratin genes in the ear skin of mixed background WT (n=7) or *Stat3* ^ΔK5^ (KO, n=7) mice bred under specific pathogen-free (SPF) conditions. The blue and red lines indicate means for WT and KO, respectively. *p < 0.05, **p < 0.01.

**Figure 3:**
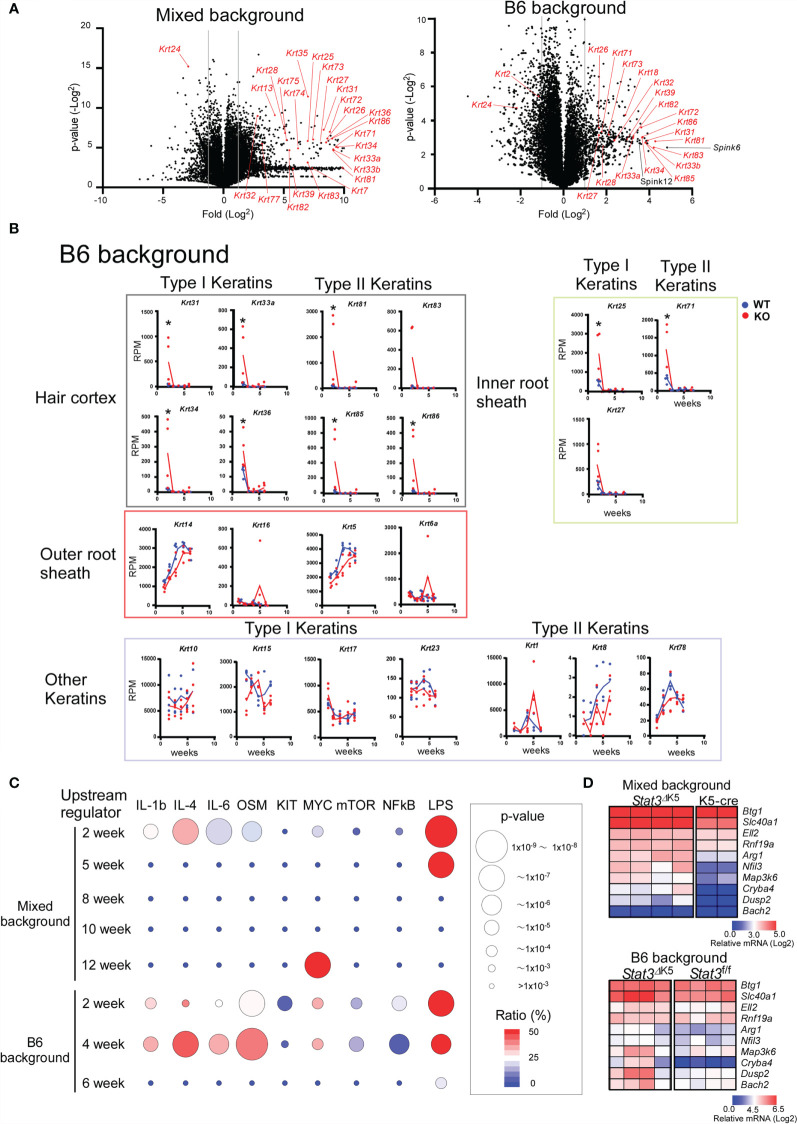
Transition of gene expression of hair follicle (HF)-specific keratins in ear skin from *Stat3* ^ΔK5^ mice. **(A)** Volcano plots showing changes in gene expression due to *Stat3* deficiency in skin of 2-weeks-old mixed (left) or B6 background (right) mice. Keratin genes are indicated with red. **(B)** The expression profiles of HF- and non-HF keratins in the ear skin of B6 background *Stat3* ^f/f^ (WT, n=4) or *Stat3* ^ΔK5^ (KO, n=4) mice bred under specific pathogen-free (SPF) conditions. The blue and red lines indicate means for WT and KO, respectively. **(C)** The results of upstream analysis of differentially expressed genes (DEGs) of mixed or B6 mouse ear skin samples at indicated weeks of age using Ingenuity Pathway Analysis (IPA) are shown by p-values and gene coverage ratios for IL-1b, IL-4, IL-6, oncostatin M (OSM), c-Kit (KIT), c-Myc (MYC), mechanistic target of rapamycin kinase (mTOR), nuclear factor-kappa B (NFkB), and lipopolysaccharide (LPS) as upstream regulators. **(D)** Heatmaps show the expression patterns of genes shown to be under the control of LPS by the upstream analysis **(C)** in the skin of 2-week-old mixed background (top) and B6 background (bottom) *Stat3* deficient (n=4) and sufficient (mixed background, n=4; B6 background, n=2) mice. *p<0.05.

**Figure 4:**
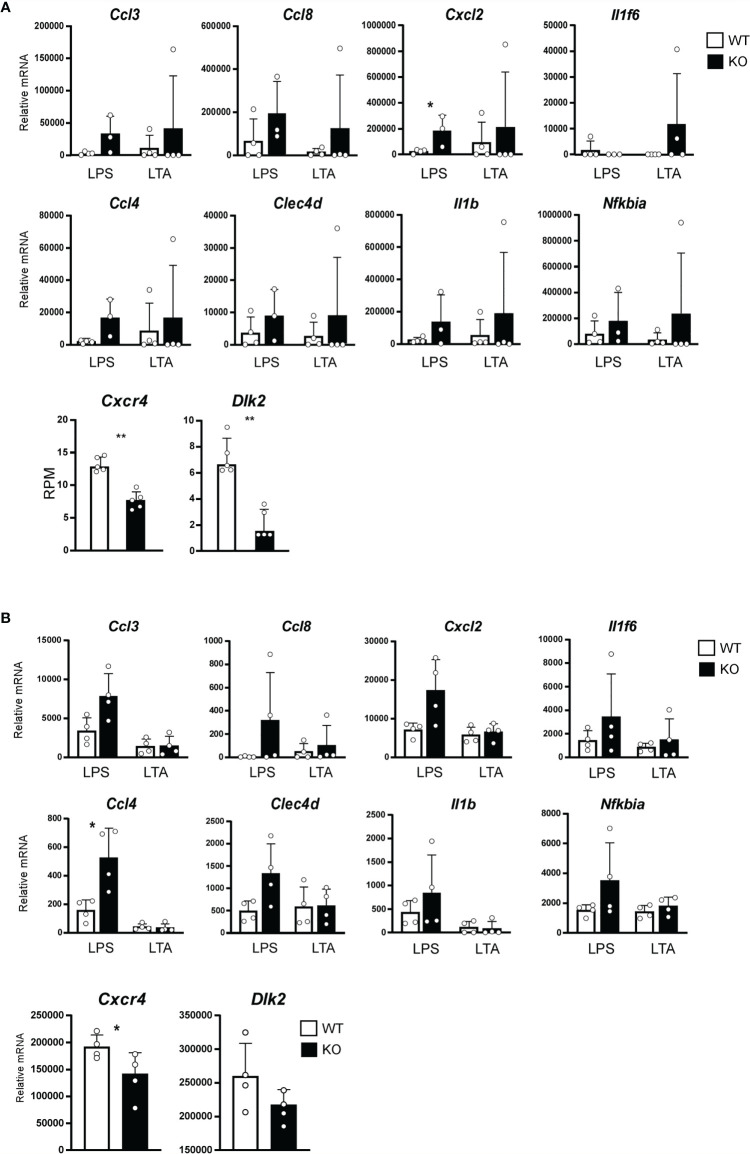
Expression of Toll-like receptor (TLR) target genes affected by the STAT3 signaling. **(A)** Ears of 6-week-old WT (white bars) or B6 background *Stat3* ^f/f^ K5-cre (KO, black bars) mice were treated with 10 mg of lipopolysaccharide (LPS) or lipoteichoic acid (LTA) continuously once a day for 10 days. Relative expression of mRNA of indicated genes that were regulated by TLR signaling was determined by a RT-qPCR (n=4, top and middle line). Total RNA were isolated from skin of 2-week-old B6 background *Stat3* ^f/f^ (WT, white bars) or *Stat3* ^f/f^ K5-cre (KO, black bars) mice, and relative mRNA expression of *Cxcr4* and *Dlk2* genes was determined by a RNA-seq (n=5, bottom line). **(B)** Keratinocytes isolated from neonatal B6 background *Stat3* ^f/f^ (WT, white bars) or *Stat3* ^f/f^ K5-cre (KO, black bars) mice were treated with 10 µg/ml of LPS or LTA for 24 hr. Relative expression of mRNA of indicated genes that were regulated by TLR signaling was determined by a RT-qPCR (n=4, top and middle line). Relative expression of mRNA of *Cxcr4* and *Dlk2* genes in keratinocytes isolated from neonatal B6 background *Stat3* ^f/f^ (WT, white bars) or *Stat3* ^f/f^ K5-cre (KO, black bars) mice was determined by a RT-q-PCR (n=4, bottom line). *p<0.05, **p<0.01.

**Figure 5:**
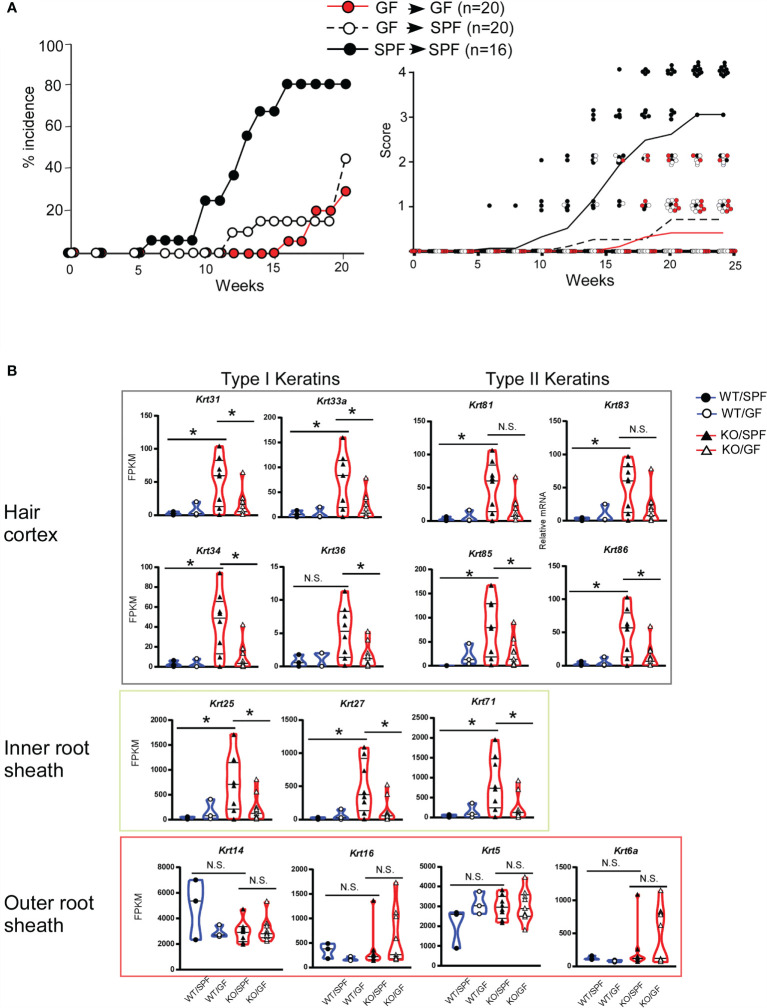
Gene expression profiles of hair follicle (HF)-specific keratin in ear skin from *Stat3* ^ΔK5^ mice in germ-free (GF) conditions. **(A)** The effect of changing the breeding environment on dermatitis onset was evaluated under specific-pathogen free (SPF) and GF conditions. Mixed background *Stat3* ^ΔK5^ (KO) mice born in GF conditions were divided into two groups at 4 weeks of age and maintained in either SPF (white dots) or GF (red dots) conditions. Black dots show data from KO mice raised in SPF conditions for life. The data indicate the percent incidence of dermatitis at the indicated weeks of age (left). The clinical scores for GF (red dots and line, n=20), GF-SPF (white dots and black dotted line, n=20), or SPF (black dots and solid line, n=16) conditions were analyzed in mixed-background *Stat3* ^ΔK5^ mice at the indicated time points (right). Lines indicate means of clinical scores in each group. **(B)** The expression profiles of HF-specific keratins in the ear skin of 20-week-old WT (circles) or KO (triangles) mice raised on SPF or GF conditions. The blue and red lines in the violin plots indicate WT and KO, respectively, and closed and open marks indicate SPF and GF, respectively. *p<0.05, N.S. indicates not significant.

We collected ear samples at different time points, specifically 2–3, 5, 8, 10, and 12 weeks of age for the mixed mice (**Figure 2**) and 2, 3, 4, 5, and 6 weeks of age for B6 mice (**Figure 3**). We performed time-course RNA-sequencing (RNA-seq) analysis and defined differentially expressed genes (DEGs) based on the criteria of ≥1.2-fold change and a false discovery rate <0.05. Based on these DEG profiles over time, the DEGs in the analysis of mixed background mice were divided into several clusters. Cluster I contained 311 DEGs that were highly expressed in KO-H and KO-D mice (**Figure 2A**). In contrast, cluster II was the largest cluster, containing 1181 DEGs exhibiting higher mRNA levels in the KO-D than in the KO-H and Control K5-cre mice. These DEGs contained several inflammatory genes associated with Toll-like receptor (TLR) -NFκB pathways (**Figure 2A**). In the present study, we focused on gene cluster I, which contained 82 keratin-related genes, to investigate the role of STAT3 in disease onset (**Figure 2A**).

Gene ontogeny by Ingenuity Pathway Analysis (IPA) of the cluster I DEGs indicated that *Stat3* deficiency in keratinocytes is associated with tissue and organ development and tissue morphology, including hair and skin development and function (**Figure 2B**). Interestingly, these keratin genes contained several HF-specific keratins, namely type I (*Krt25-28, 31-36*, and *39-40*) and type II (*Krt71*, *73-75, 81-84*, and *86*) (**Figure 2A**). The HF-keratin genes were highly expressed only in 2–3-week-old KO mice and reduced to the levels observed in control mice at 5 to 10 weeks. Interestingly, the expression of these genes was again increased in mixed KO-D mice after 12 weeks (**Figure 2C**). A similar oscillating expression pattern for the HF-keratin genes was found in the B6 KO mice, although the increase was more prominent in the mixed mice (**Figures 3A, B**). Other epithelial keratins, such as *Krt14* and *Krt6*, expressed in the outer root sheath of HFs but not in hair epithelial cells, were normally expressed (**Figures 2C**, **3B**). Notably, gene expression of *Krt15*, a marker gene for HF stem cells (28), at 2 weeks of age was similar between KO and WT mice of both genetic backgrounds (**Figures 2C** and **3B**). These results indicate that *Stat3* deficiency in epithelial cells leads to the dysregulation of HF-keratin genes involved in epithelial regeneration. An analysis of upstream regulators in the positive transcriptome from the skin of mixed and B6-background mice using IPA indicated STAT3-related inflammatory cytokines, including IL-6 and oncostatin M (OSM) (**Figure 3C**). Notably, lipopolysaccharide (LPS) was shown to be an upstream signal of DEGs that were upregulated in the infant skin of KO mice (**Figures 3C, D**). To investigate the effect of STAT3 signaling on TLR signaling, we applied TLR agonists, LPS or lipoteichoic acid (LTA), to the skin of KO mice and examined the expression of genes downstream of TLR signaling. We found that the treatment of LPS increased the expression of *Cxcl2*, which is a TLR signal target gene in the skin of 6-week-old B6 KO mice (**Figure 4A**). Furthermore, the other genes downstream of TLR signaling, including *Ccl3*, *Ccl4*, *Ccl8*, *Clec4d*, *Il1b*, *Il1f6*, and *Nfkbia* tended to be upregulated by LPS and LTA stimulation in the skin of B6 KO mice (**Figure 4A**).

To investigate how STAT3 signaling affects TLR signaling, we analyzed RNA-seq data from the skin of 2-week-old B6 KO mice. We found that STAT3 signaling increased the expression of *Dlk2* and *Cxcr4*, which are known as inhibitors of TLR signal (**Figure 4A**) (29, 30). Similar changes of gene expression were found in keratinocytes isolated from neonatal B6 KO mice stimulated with LPS or LTA *in vitro* (**Figure 4B**). These results indicate that STAT3 signaling negatively regulates TLR-mediated inflammatory responses in infant skin.

### The Skin Microorganism Composition in the Infant Period Determines the Onset of Dermatitis

Folliculitis is a skin disorder commonly observed in patients with HIES, and HF dysbiosis is associated with inflammation (31–33). However, it remains unclear how STAT3 regulates skin immune homeostasis. We speculated that a bacterial component induces STAT3 activation associated with skin homeostasis and disease onset. To examine the effect of the bacterial components in the feeding period, we compared disease onset between germ-free (GF) and SPF mice. The same litters of *Stat3* ^ΔK5^ mice were born and maintained in GF conditions by 4 weeks of age, after which half of the mice were transferred to SPF conditions and the other half were maintained under GF conditions. The SPF group consistently developed dermatitis, whereas the GF group had a lower disease incidence (**Figure 5A**, left). Interestingly, disease onset in the SPF group that had been transferred from GF conditions was significantly delayed compared to that in the SPF mice (**Figure 5A**, right).

We then compared the expression levels of HF-related keratin genes in mice under SPF and GF conditions. The expression levels of keratin genes in the *Stat3* ^ΔK5^ skin were markedly increased in mice under SPF conditions but not in GF conditions (**Figure 5B**). The same expression pattern of HF-keratin genes in mice under GF-to-SPF conditions was observed in *Stat3*-sufficient skin. These results indicate that the microorganism composition in infant skin determines the lifetime onset of dermatitis through the STAT3-mediated regulation of HF-related keratin genes.

### *Stat3* Deficiency in the Skin Leads to the Dysregulation of Epidermal Development

**Figure 6:**
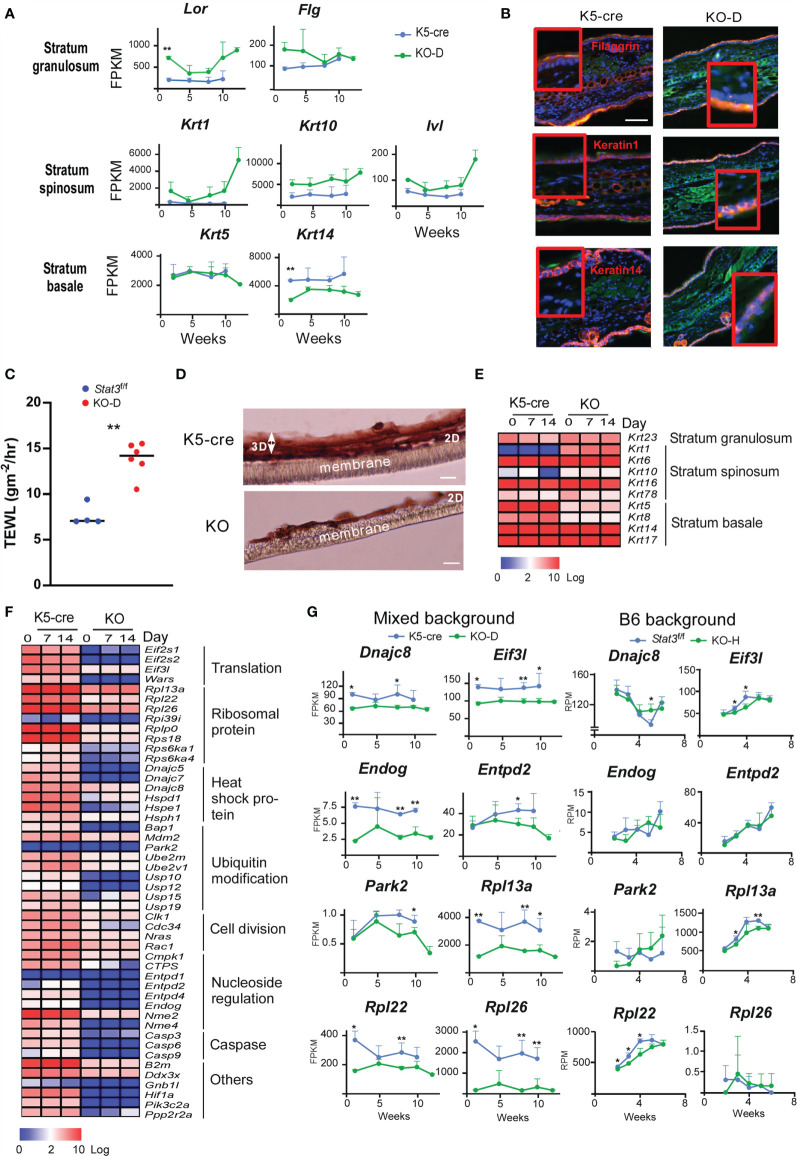
Dysregulation of epidermal development in *in vitro* cultured *Stat3* ^ΔK5^ keratinocytes. **(A)** Time-dependent changes in *in vivo* mRNA expression of *Lor*, *Flg*, *Krt1*, *Krt10*, *Ivl*, *Krt5*, and *Krt14* are shown as lines for K5-cre (blue), and KO-D (green) mice. **(B)** Histological distribution of keratin 1 and 14, and filaggrin protein in ear skin of WT and KO-D mice. Green and blue colors show Ki-67 expression and nuclei, respectively. **(C)** Transepidermal water loss (TEWL) from the ear of mixed background *Stat3^f/f^* (n=4) or *Stat3* ^ΔK5^ (n=5) mice was measured at 16 weeks of age. **(D)** Hematoxylin and eosin staining of 3D-cultured keratinocytes (day 14). Keratinocytes were isolated from mixed background *Stat3*-sufficient (K5-cre) or deficient (KO) newborn mice. After 3 days of culture in liquid medium (2D-culture), keratinocytes were induced to undergo 3D skin structure formation by exposing the apical layer to air, and cells were cultured for another 7 or 14 days. **(E)** Heatmap depicting *in vitro* keratin gene expression in 2D (day 0) and 3D (day 7 and 14) cultures. **(F)** Heatmap depicting *in vitro* cell proliferation-related gene expression in 2D (day 0) and 3D (day 7 and 14) cultures. **(G)** Time-dependent changes in gene expression of *Dnajc8*, *Eif3l*, *Endog*, *Entpd2*, *Park2*, *Rpl13a*, *Rpl22*, and *Rpl26* in the ear skin of mixed background (left) or B6 background (right) *Stat3*-sufficient (K5-cre for mixed background, *Stat3* ^f/f^ for B6 background) and deficient (KO) mice are shown as lines for *Stat3*-sufficient (blue, n=4) and *Stat3*-deficient (KO, green, n=4) mice. Scale bar = 100 μm. *p<0.05, **p<0.01.

The dermatitis onset observed in *Stat3* ^ΔK5^ skin depended on the dysregulation of HF-keratin gene expression. Thus, we next investigated how the defect in STAT3 signaling impacts the skin structure that leads to dermatitis. *Stat3-*deficient skin showed higher mRNA expression of *Krt1* than that in *Stat3*-sufficient mice of the same age, whereas a marker of the stratum basale, *Krt14*, was downregulated (**Figures 6A, B**). *Lor* and *Flg* expression in the stratum granulosum was upregulated in *Stat3-*deficient skin, possibly due to compensatory barrier dysfunction (**Figures 6A, B**). Next, we measured transepidermal water loss (TEWL), which represents the evaporation rate of water from the dermal skin, in the skin of KO mice to directly investigate the role of STAT3 signaling in maintaining skin barrier function. The results showed an elevation in the TEWL value, which indicates an increase in water evaporation from the dermis, indicating that *Stat3* signaling is necessary to maintain the barrier function of the skin (**Figure 6C**).

To further understand the role of STAT3 in epidermis formation, we established a three-dimensional (3D) skin culture system using primary keratinocytes obtained from newborn skin. The *Stat3* ^ΔK5^ keratinocytes had a normal two-dimensional layer but exhibited growth retardation in the 3D phase (**Figure 6D**). RNA-Seq analyses indicated that the mRNA expression of *Krt1* and *Krt10* genes expressed in stratum spinosum was increased in *Stat3* ^ΔK5^ keratinocytes *in vitro* (**Figure 6E**), which is consistent with the *in vivo* results, despite that changes in the mRNA expression of *Krt5* and *Krt14* in the stratum basale differed between *in vivo* and *in vitro*. To prove the possibility that an imbalance of paired keratins influences skin structure, we examined how the forced expression of *Krt1* or *Krt10*, which forms a dimer, affects morphological change and cell survival in epithelial cells. The results indicated that imbalance in keratin dimer expression leads to defect in skin structure and cell survival (**Supplementary Figure S1**). Furthermore, the mRNA expression of the initiation components of eukaryotic translation, EIF3L, 60S ribosomal subunits, RPL13a, 22, and 26, HSP40 family proteins, and DNAJC 8 were downregulated both *in vivo* and *in vitro* in *Stat3* ^ΔK5^ keratinocytes (**Figures 6F, G**). These results indicate that STAT3 is essential for protein synthesis and metabolic pathways in keratinocyte differentiation (34, 35). Therefore, we speculate that a defect in STAT3 significantly impacts skin structure and barrier function *via* the dysregulation of epithelial regeneration.

## Discussion

The present study indicates that STAT3-dependent gene expression in keratinocytes plays an essential role in regulating skin homeostasis. STAT3 signaling in skin epithelial cells is critical for controlling the expression of HF-specific keratin genes in infant skin. The excess expression of HF-specific keratin genes depends on the presence of skin microorganisms, indicating that the STAT3 signaling activated by skin microorganisms plays a crucial role in regulating the expression of HF-specific keratin genes. The excess expression of HF-specific keratin genes during the infant period was correlated with skin barrier dysfunction and the development of dermatitis, suggesting that STAT3 signaling in infant skin is crucial for maintaining skin barrier homeostasis to prevent dermatitis.

We demonstrated that skin-specific *Stat3* deficiency caused oscillating expression of HF-specific type I and type II keratin genes in early life stages. These results suggest that the polymerization of keratin is impaired in the process of heterodimer formation mediated by the pairing of type I and type II keratin proteins. This altreated expression is closely associated with increased rates of water evaporation and dermatitis development, suggesting the importance of epithelial homeostasis and maintenance of the skin barrier. We demonstrated that the forced expression of *Krt1* or *Krt10*, which forms a dimer, affects viability of skin epithelial cells, suggesting that Keratin gene expression, balanced by STAT3 signaling, is important for cell survival (**Supplementary Figure S1**). Consistent with our results, transgenic expression of Keratin 8 or 16 proteins has been reported to cause hyperkeratotic and developmental defects (36, 37). Further, mutations in hair keratins or hair follicle-specific epithelial keratins (Krt 81, 83, and 86) cause skin disorders, including epidermolytic hyperkeratosis and ichthyosis bullosa of siemens (38).

STAT3 signaling has been reported to contribute to epithelial differentiation, proliferation, and carcinogenesis (39–41). The loss of function of STAT3 in keratinocytes increases the number of apoptotic HFSCs and impairs epidermis regeneration and the hair cycle process (19, 23). Meanwhile, gain of function increases HFSCs and progenitor cells above the bulge region (24). These observations support our notion that STAT3 signaling is an essential epithelial component that negatively controls type I and type II keratin expression to maintain skin homeostasis and barrier formation. However, further investigation is necessary to elucidate how the dysregulation of HF-specific keratin genes impacts dermal structure and barrier functions.

Interestingly, the transient increase in HF-specific keratin only occurred in SPF conditions but not in GF conditions, suggesting that bacterial components control the STAT3-mediated transient keratin expression in the skin. **Figure 3C** shows the transient activation of TLR signals at 2–5 weeks of age. In patients with scarring alopecia, apoptosis of bulge HFSCs is linked to decreased expression of *Krt15*, a key marker for bulge stem cells (42, 43). Dysbiosis occurring in the *Sox9*-expressing tissue specific deficiency of ADAM17 results in disrupted HF development (44), indicating the importance of commensal bacteria in HF homeostasis. Therefore, it is reasonable to speculate that the interplay between STAT3 signaling and commensal bacteria-derived TLR signaling might contribute to type I and type II HF-specific keratin expression during the infant stage. This transient increase in HF-specific keratin might impact the skin barrier function and microbiome later in life. In fact, skin-specific *Stat3*-deficient mice showing a transient increase in keratin gene expression exhibit TLR-mediated NF-κB activation followed by dermatitis onset. Thus, the interplay between STAT3 signaling and commensal bacteria might affect penetration of the skin barrier and the susceptibility of immune responses to invading microbes.

It is unclear why the loss of STAT3 signaling causes abnormal keratin expression only in infant skin. Since most patients with atopic dermatitis develop the disease in early childhood and show symptom relief as they grow up, STAT3 signaling may play a particularly important role as a defense against bacteria in the immature skin immune environment of early childhood. Further studies are needed to verify the role of STAT3 in infant skin.

This report found that STAT3 signaling negatively regulates the expression of genes controlled by the TLR pathway in skin epithelial cells. Crosstalk between JAK/STAT and TLR pathways has been suggested in several reports (45–47).　In the case of macrophages, STAT3 has been reported to be a negative regulator for TLR4 signaling (47). Therefore, we concluded that STAT3 signaling plays a role in negatively regulating the inflammatory genes downstream of the TLR pathway in skin epithelial cells. Penetration of skin commensal bacteria increases the risk of the susceptibility of inflammatory responses to invading microbes. Therefore, it is assumed that the STAT3-mediated negative regulation may effectively prevent excessive inflammatory responses in the neonatal period. Further studies are needed to elucidate the molecular mechanism of how STAT3 influences the TLR pathway as the specific negative regulator. However, this is a critical aspect of controlling skin homeostasis, preventing inflammatory responses.

Taken together, this study indicated that STAT3 signaling in infant skin is essential for the maintenance of healthy skin homeostasis and regulates the HF development that is disturbed by resident skin microorganisms to maintain the integrity of skin barrier functions. Therefore, we conclude that STAT3 signaling in skin epithelial cells plays an essential role in skin homeostasis. These results suggest the potential of antimicrobial therapy in preventing the disruption of skin homeostasis caused by STAT3 signaling failure.

## Methods

### Mice

The generation of K5-cre and *Stat3* ^f/f^ mice has been previously described (16, 20). The mixed background of *Stat3* ^f/f^ K5-cre mice was maintained by intercrossing to maintain the 129 genetic background (KO). *Stat3* ^f/f^ K5-cre mice with a B6 background were generated by backcrossing with B6 mice for 12 generations (B6-KO). All mice used in this study were maintained under SPF or GF conditions as indicated, and animal care was performed in accordance with the guidelines of the RIKEN Yokohama Institute.

### Antibodies

Rabbit anti-Filaggrin (905804), rabbit anti-Keratin-1 (905204), rabbit anti- Keratin-14 (905304), and rat anti-Ki-67 (11F6, 151202) antibodies were purchased from BioLegend (San Diego, CA, USA). Donkey anti-rabbit IgG Cy3 (Jackson ImmunoResearch, West Grove, PA) and goat anti-rat IgG Alexa Fluor 488 (Life Technologies, Carlsbad, CA) were used as secondary antibodies for immunohistochemistry.

### Histology and Immunohistochemistry

Frozen skin sections were fixed with acetone. After blocking with 3% BSA/PBS for 30 min, the sections were incubated with primary antibodies in 1% BSA/PBS for 30 min at room temperature. The sections were washed with 0.05% tween20 in PBS and incubated with secondary antibodies in 1% BSA/PBS for 30 min at room temperature. After washing the sections with PBS for 5 min, they were mounted using Mountant Permafluor (Thermo Fisher Scientific, Waltham, MA, USA). Images were acquired using a Keyence BZ-X700 (Keyence, Osaka, Japan). For hematoxylin and eosin staining, frozen sections of ear skin or layered keratinocytes on the membrane were fixed with 4% PFA in PBS and stained with hematoxylin and eosin (Muto Chemical, Tokyo, Japan). Images were acquired with a Keyence BZ-X700 (Keyence).

### Generation and Analysis of Dynamic Gene Expression Profiles

Total RNA was isolated with TRIzol (Thermo Fisher Scientific) from mouse ears obtained at different time points, specifically 2–3, 5, 8, 10, and 12 weeks of age, and kinetic RNA sequence analysis was carried out. cDNA was synthesized using an NEBNext Ultra RNA Library Prep Kit for Illumina (NEB Biolabs, Inc., Ipswich, MA, USA) according to the manufacturer’s instructions. Sequencing data were obtained using the HiSeq 1000 system (Illumina, San Diego, CA, USA), which reads a 50 bp sequence (single-end 50 bp pair reads). Hierarchical clustering and heat mapping of data were performed with MeV (48). Data were analyzed with Strand NGS (Strand Genomics, San Francisco, CA, USA) and IPA (Ingenuity, Redwood City, CA) (49). RNA-Seq reads were first aligned to the mouse genome (mm9) using ‘TopHat2’ (50). The raw gene counts were then normalized and expressed as frequency per kilobase per million mapped reads (FPKM) for 20,628 genes annotated in the reference genome database.

### 3′ mRNA-Seq Analysis

Total RNA was isolated with TRIzol from mouse ears obtained at different time points, specifically 2, 3, 4, 5, and 6 weeks of age, and kinetic RNA sequence analysis was carried out. The Lexogen QuantSeq 3′ mRNA-seq library prep kit (Lexogen GmbH, Vienna, Austria) was used with 10 ng of total RNA from the ear skin. Sequencing data were obtained using the HiSeq 1000 system, which reads a 50 bp sequence (single-end 50-base pair reads). Sequenced reads were trimmed for adaptor sequences, masked for low-complexity or low-quality sequences, and then mapped to the whole mouse genome using STAR 2.7.0c with the mouse genome (mm10 GRCm38.p6 ENSEMBL GTF: GRCm38.97 ENSEMBL). Raw gene counts were normalized and expressed as reads per million mapped reads (RPM).

### BioMark HD Gene Expression System

Total RNA was isolated with TRIzol from ear samples, and reverse transcription and pre-amplification were performed with Reverse Transcription Master Mix and Preamp Master Mix (Fluidigm, South San Francisco, CA, USA), respectively. Quantitative real-time PCR was carried out in the Dynamic Arrays integrated fluidic circuit of a BioMarkHD system (Fluidigm) with specific primer pairs as indicated in **Supplementary Table T1**.

### RT-q-PCR

Total RNA was isolated with TRIzol from keratinocytes, and reverse transcription and pre-amplification were performed with Superscript III reverse transcriptase (Thermo Fisher Scientific). Quantitative real-time PCR was carried out using SYBR Premix Ex Taq II (Takara Bio Inc, Shiga, Japan) with specific primer pairs as indicated in **Supplementary Table T1**.

### cDNA Cloning and Plasmid Construction

Total RNA was isolated from the back skin of 8-week-old female C57BL6/J mice using an RNeasy Fibrous Kit (Qiagen, Hilden, Germany). First-strand cDNA was prepared from total RNA by Superscript II reverse transcriptase (Thermo Fisher Scientific). The DNA fragment encoding the open reading frame of mouse keratin 1 or mouse keratin 10 were amplified from mouse back skin cDNA by PCR using following primer sets (keratin 1, AATTGGTACCATGAGTCTACAGTGTAGCTCCAGGTCCCTG/ATATCCCGGGTTATTTGGTCCCTCGGGAGTAACTGGTGG; keratin 10, AATT-GGTACCATGTCTGTTCTATACAGCTCCAGCAGCAAG/ATATGATATC-TTAGTATCTTGGTCCTTTAGATGATTGGTC) primers. The purified PCR product was digested with KpnI and SmaI and subcloned into the pmCherry-c1 vector was purchased from Takara Bio Inc. (Shiga, Japan).

### TWEL Analysis

TWEL was measured in the ears of 16-week-old mice using a VAPO SCAN (ASCH JAPAN, Tokyo, Japan). Five measurements were taken, and the mean of the three medians was used as the measurement value.

### 3D Epidermal Cell Culture

Primary keratinocytes were obtained from the epidermis isolated from neonatal mice. The epidermis was separated from the dermis following overnight incubation at 4°C in 5 mg/mL dispase (Roche, Basel, Switzerland) in CnT-PCT medium (CELLnTEC, Advanced Cell Systems AG, Bern, Switzerland). Keratinocytes were suspended using TrypLE (CELLnTEC) and seeded on Millicell PCF inserts (Millipore, Billerica, MA) in a 60 mm cell culture dish containing CnT-PCT medium. After 3 days of culture in a humidified incubator at 37°C with 5% CO_2_, the CnT-PCT medium was replaced with 3D Prime differentiation medium (CELLnTEC) and keratinocytes were cultured for 16 h at 37°C with 5% CO_2_. To initiate 3D structure formation, the medium inside the insert was removed, and keratinocytes were maintained by changing the outside medium every 3 days. After 7 or 14 days of culture, cells were harvested and RNA was extracted using an RNAeasy Mini Kit (Qiagen, Hilden, Germany) for RNA-seq analysis.

### Bioinformatics Analysis

For clustering, 2,064 DEGs (p<0.05, FKPM>2.0) were extracted by comparing the expression values at each week. The analysis was implemented by Ward’s method for hierarchical clustering based on Pearson’s correlation coefficient with the function ‘heatmap.2’ in R software (R version 3.2.2, R Core Team (2017), R Foundation for Statistical Computing, Vienna, Austria). The heatmap was normalized using z-scores. Upstream molecules were predicted by upstream analysis of IPA (Ingenuity, Redwood City, CA, USA).

### Statistical Analysis

Unless otherwise stated, all statistical analyses were performed using the Mann-Whitney test.

## Data Availability Statement

The data presented in the study are deposited in the GEO repository, accession number GSE86071, GSE185340, and GSE185585.

## Ethics Statement

The animal study was reviewed and approved by RIKEN Yokohama Institute animal experimental committee.

## Author Contributions

MK designed and conceptualized the research and YS, KM, and HK performed mouse experiments. SK, KM, and OO performed transcriptome analysis. SK, TM, and KM performed immunohistostaining. MU, KI, RT, and MO-H performed bioinformatics analysis. WS, YI, and KH performed microbiological analysis. TM and KM performed *in vitro* experiments using keratinocytes. KM and MK prepared the manuscript. All authors contributed to the article and approved the submitted version.

## Conflict of Interest

The authors declare that the research was conducted in the absence of any commercial or financial relationships that could be construed as a potential conflict of interest.

## Publisher’s Note

All claims expressed in this article are solely those of the authors and do not necessarily represent those of their affiliated organizations, or those of the publisher, the editors and the reviewers. Any product that may be evaluated in this article, or claim that may be made by its manufacturer, is not guaranteed or endorsed by the publisher.

## Supplementary Material

Supplementary Figure 1 Keratinocytes were freshly isolated from neonatal B6 background mice, and were transfected by TransIT-Keratinocyte Transfection Reagent (Mirus Bio, Madison, WI, USA) with monomeric Cherry (as a control, white dots), Keratin 1-Cherry (black dots) or Keratin 10-Cherry (red dots) expression plasmids. Twenty-four hours after the transfection, picture of cells were obtained with a BZ-X800 microscopy system (Keyence). In the pictures on top, white arrow heads and black arrow heads indicate normal morphology keratinocytes and Keratin 1 or 10-Cherry expressing-rounding keratinocytes, respectively. Cell number of live or dead keratinocytes with or without Cherry fluoresces in a 30000 µm^2^ square field was counted (n=5 to 7) and shown in the dot plot on bottom. Bars indicates mean. **p<0.01.

Role of STAT3 signaling in maintaining homeostasis of skin keratinocytes. A schematic diagram shows that STAT3 signaling is required for balancing type I and II *Keratin* genes expression disturbed by microorganisms to maintain skin homeostasis.

Supplementary Table 1 List of primer sets for RT-q-PCR analysis.
