# Supplementary material for: Essential Role of STAT3 Signaling in Hair Follicle Homeostasis
Source: Front Immunol. 2021 Nov 11;12:663177. doi: 10.3389/fimmu.2021.663177 (PMC8635990; doi:10.3389/fimmu.2021.663177)
Supplement: Supplementary Figure 1 — Keratinocytes were freshly isolated from neonatal B6 background mice, and were transfected by TransIT-Keratinocyte Transfection Reagent (Mirus Bio, Madison, WI, USA) with monomeric Cherry (as a control, white dots), Keratin 1-Cherry (black dots) or Keratin 10-Cherry (red dots) expression plasmids. Twenty-four hours after the transfection, picture of cells were obtained with a BZ-X800 microscopy system (Keyence). In the pictures on top, white arrow heads and black arrow heads indicate normal morphology keratinocytes and Keratin 1 or 10-Cherry expressing-rounding keratinocytes, respectively. Cell number of live or dead keratinocytes with or without Cherry fluoresces in a 30000 µm2 square field was counted (n=5 to 7) and shown in the dot plot on bottom. Bars indicates mean. **p<0.01. [file DataSheet_1.pdf]

Supplementary Figure S1

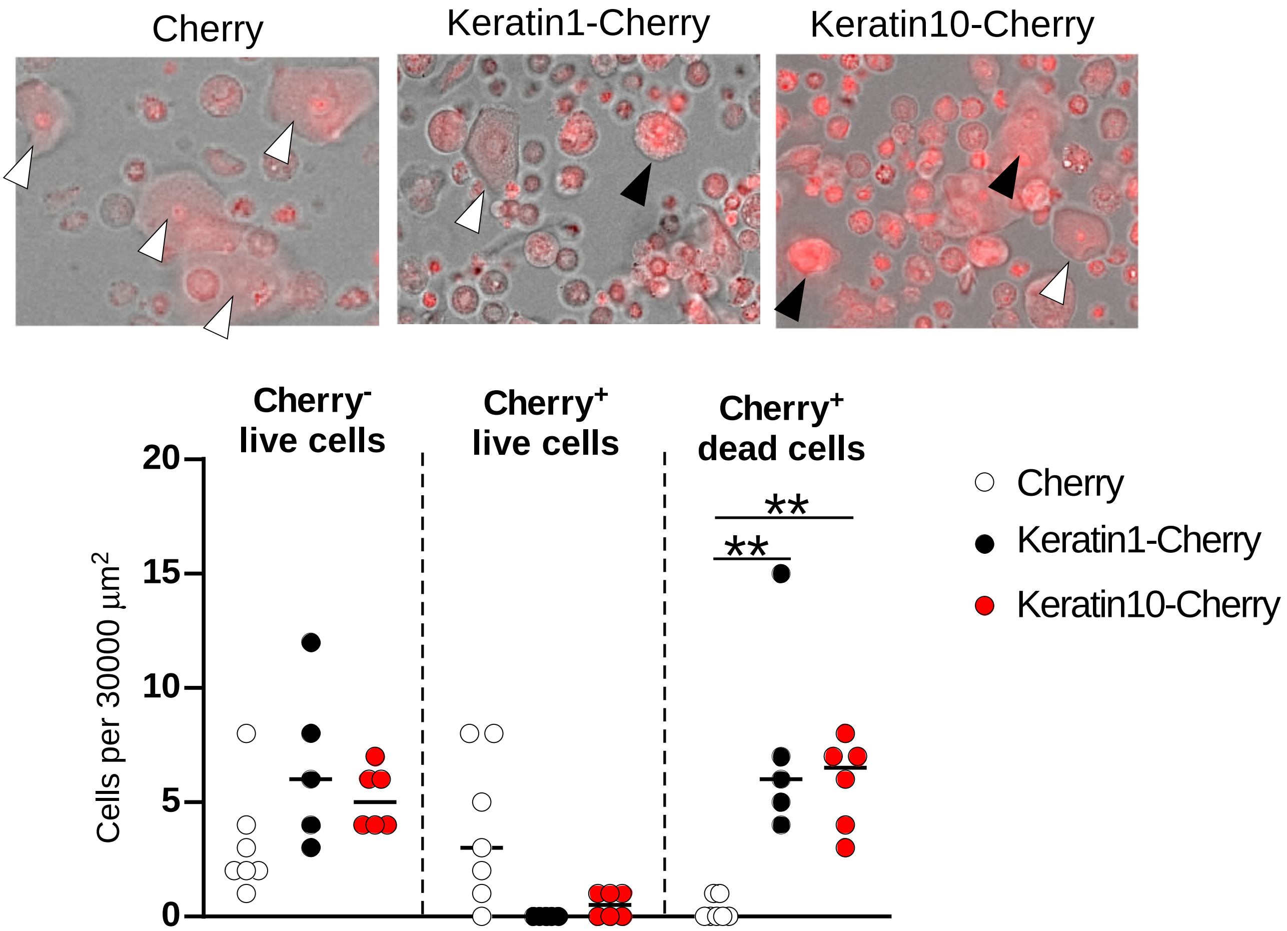

Supplementary Figure S2

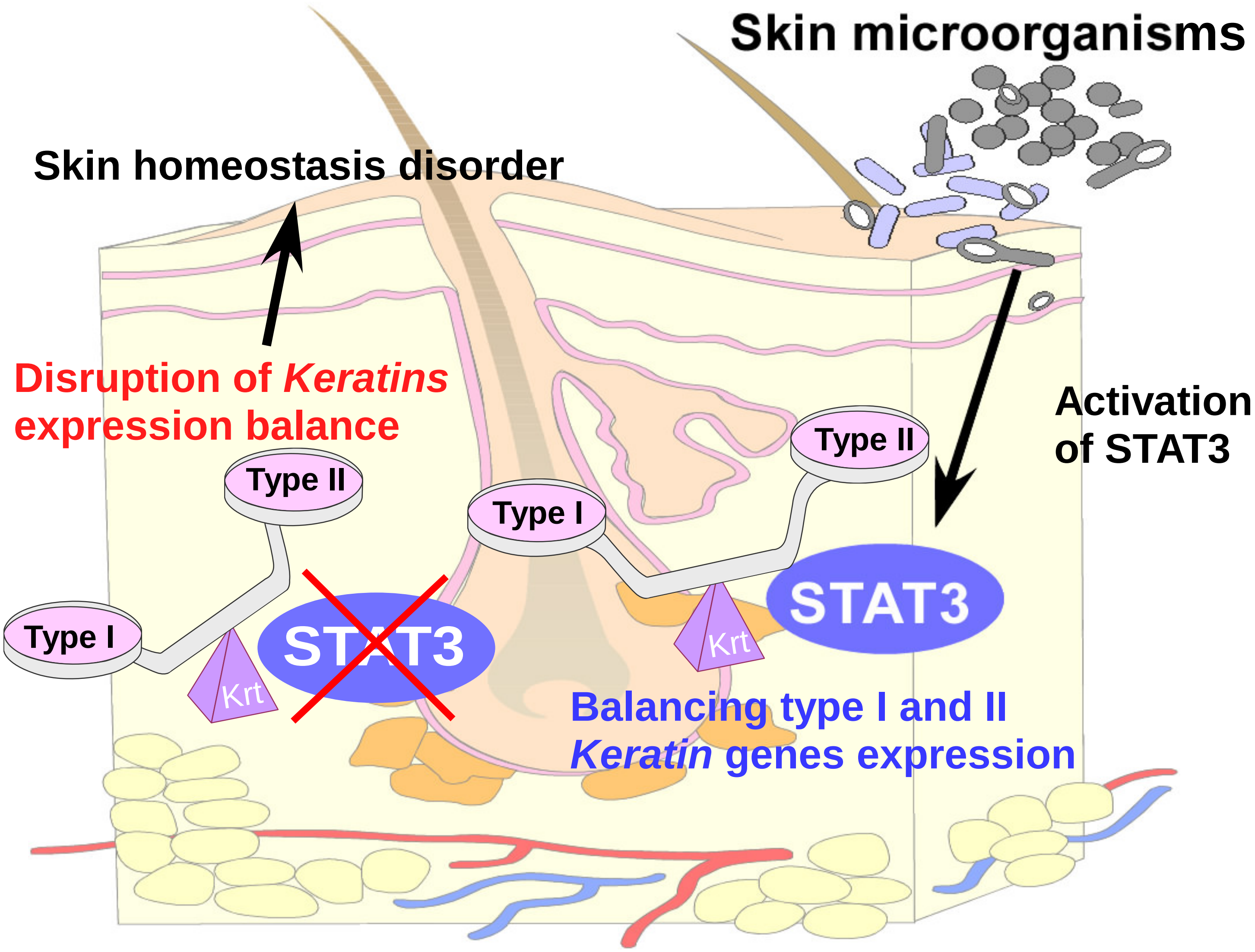

Supplementary Table T1

Gene symbol	Forward	Reverse
Actb:	5'-ccctaaggccaaccgtgaaa-3'	5'-agcctggatggctacgtaca-3'
Ccl3:	5'-accatgacactctgcaacca-3	5'-gaatcttccggctgtaggagaa-3'
Ccl4:	5'-ctgtgctccagggttctca-3'	5'-agcaaagactgctggtctca-3'
Ccl8:	5'-aatatccagtgccccatggaa-3'	5'-cacttctgtgtggggtctaca-3'
Clec4d:	5'-cacgagagtaacgtgcatcc-3'	5'-taacaggacagcaggtccaa-3'
Cxcl2:	5'-cccctggttcagaaaatcatcc-3'	5' tcctttccaggtcagttagcc-3'
Il1b:	5'-tggcaactgttcctgaactca-3'	5'-gggtccgtcaacttcaaagaac-3'
Il1f6:	5'-tgtgtggatcctgcagaaca-3'	5'-attggcatgggagcaaggta-3'
Nfkbia:	5'-gagcgaggatgaggagagcta-3'	5'-ggcctccaaacacacagtca-3'
Cxcr4:	5'- gactggcatagtcggcaatg-3'	5'- agaaggggagtgtgatgacaaa-3'
Dlk2:	5'- gccctcaacttcacatgcc-3'	5'- cgcatcagacagtcgtccac-3'
